# Differences in the distribution of triggers among resting state networks in patients with juvenile myoclonic epilepsy explained by network analysis

**DOI:** 10.3389/fnins.2023.1214687

**Published:** 2023-10-04

**Authors:** Dadong Luo, Yaqing Liu, Ningning Zhang, Tiancheng Wang

**Affiliations:** ^1^Department of Neurology, Lanzhou University Second Hospital, Lanzhou, China; ^2^Second School of Clinical Medicine, Lanzhou University, Lanzhou, China

**Keywords:** juvenile myoclonic epilepsy, systematic mapping, functional imaging, network analysis, seizure

## Abstract

**Background:**

Juvenile myoclonus epilepsy (JME) is an idiopathic generalized epilepsy syndrome. Functional connectivity studies based on graph theory have demonstrated changes in functional connectivity among different brain regions in patients with JME and healthy controls. However, previous studies have not been able to clarify why visual stimulation or increased cognitive load induces epilepsy symptoms in only some patients with JME.

**Methods:**

This study constructed a small-world network for the visualization of functional connectivity of brain regions in patients with JME, based on system mapping. We used the node reduction method repeatedly to identify the core nodes of the resting brain network of patients with JME. Thereafter, a functional connectivity network of the core brain regions in patients with JME was established, and it was analyzed manually with white matter tracks restriction to explain the differences in symptom distribution in patients with JME.

**Results:**

Patients with JME had 21 different functional connections in their resting state, and no significant differences in their distribution were noted. The thalamus, cerebellum, basal ganglia, supplementary motor area, visual cortex, and prefrontal lobe were the core brain regions that comprised the functional connectivity network in patients with JME during their resting state. The betweenness centrality of the prefrontal lobe and the visual cortex in the core functional connectivity network of patients with JME was lower than that of the other brain regions.

**Conclusion:**

The functional connectivity and node importance of brain regions of patients with JME changed dynamically in the resting state. Abnormal discharges originating from the thalamus, cerebellum, basal ganglia, supplementary motor area, visual cortex, and prefrontal cortex are most likely to lead to seizures in patients with JME. Further, the low average value of betweenness centrality of the prefrontal and visual cortices explains why visual stimulation or increased cognitive load can induce epileptic symptoms in only some patients with JME.

## 1. Introduction

Juvenile myoclonic epilepsy (JME) is an idiopathic generalized epilepsy syndrome that has a predilection for ages from 8 to 18 years (Chawla et al., 2021). Previous clinical studies have shown that all patients with JME experience myoclonic seizures. Notably, 85% of patients have combined generalized tonic-clonic seizures (Zhang et al., 2019) and 10–15% of patients with JME have combined typical absence seizures (Camfield et al., 2014). Furthermore, approximately 30% of patients with JME are characterized by photosensitivity (Ur Özçelik et al., 2021) or decreased cognitive function (Chawla et al., 2021). The inconsistency in the symptoms indicates that the activity of various brain regions in patients with JME is not consistent. Previous studies have elucidated the association between abnormal discharges in various brain regions and epileptic symptoms. For example, it was reported that normal electrical activity in the supplementary motor area (SMA) negatively regulated myoclonic seizures in patients with JME (Caciagli et al., 2020). Further, atypically increased activity in the subcortical structures i.e., the cerebellum (Cere), basal ganglia (BG), brainstem, and thalamus (Tha) is crucial for tonic-clonic seizures. It has also been reported that absence seizures are associated with synchronization in the thalamus (Fogerson and Huguenard, 2016) and that reduction in the number of dendritic spines in the prefrontal cortex (PFC) correlated with a decline in executive capacity and emotion control (Tsypes et al., 2018). To the best of our knowledge, previous studies could neither compare the contribution of the abnormal discharges of origin in various brain regions regarding the appearance of epileptic symptoms nor explain why only some patients with JME developed seizures in response to increased light stimulation or cognitive load.

In this study, we annotated and categorized the literature by systematic mapping (SM). Thereafter, we selected the literature related to “Juvenile myoclonic epilepsy” and “functional connectivity,” and followed the subjects, research equipment, research indexes, etc., to obtain the necessary information for drawing a small-world network model of the functional connectivity of brain regions at rest in patients with JME. Thereafter, the core brain regions with altered functional connectivity in the resting state of patients with JME were screened out by the node reduction method. Finally, the core functional brain networks in the resting state of patients with JME were constructed and analyzed by tracks restriction of the main white matter fibers in the brain regions to investigate the reasons for the differences in the distribution of triggers among patients with JME in their resting state, which was resolved stepwise by the aforementioned steps.

## 2. Materials and methods

### 2.1. System mapping

#### 2.1.1. Data source

This study focused on the differences in functional connectivity between brain regions in patients with JME and healthy control groups. Particularly, the following two questions were included in the investigation:

• Do patients with JME have specific brain regions wherein substantially altered functional connectivity is concentrated?
• What are the patterns and significance of changes in the functional connectivity among the brain regions mentioned above?

**FIGURE 1:**
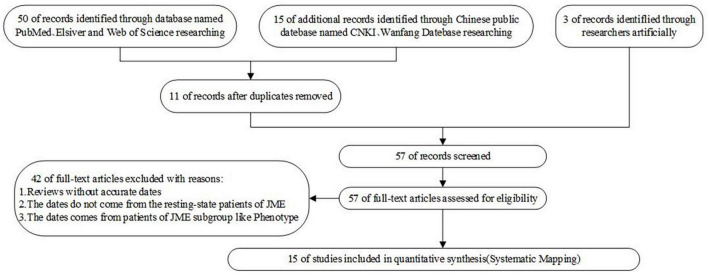
PRISMA flow diagram of the studies selection process.

**FIGURE 2:**
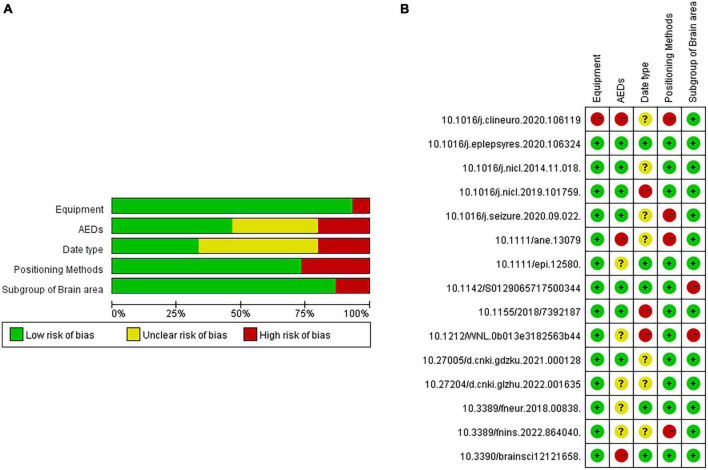
Risk analysis of bias included in the research literature. **(A)** single literature analysis. **(B)** total literature analysis.

Based on the above questions, this study selected 50 papers on PubMed, Elsevier, and Web of Science with [“Juvenile myoclonus epilepsy” or “JME”] and [“functional connectivity”] as the search methods. Further, 15 papers on CNKI and Wanfan databases were selected, with [“juvenile myoclonus epilepsy”] and [“functional connectivity”] as the search methods. In addition, three Chinese master’s and doctoral theses were manually included. After excluding duplicate papers, those with dynamic functional connectivity, those whose dates did not come from the resting state of patients with JME or whose dates came from patients in the JME subgroup, and the reviews without accurate dates, 15 studies were finally included. During the process of literature selection, the Kitchenham protocol was followed and the Review Manage tool was used to read each article. The flowchart for the literature screening is shown in Figure 1, and the risk of bias included in the research literature is shown in Figure 2.

#### 2.1.2. Indicator conversion

Two independent researchers extracted the number and demographic information of patients with JME and healthy control groups as well as the indicators that reflected the functional connectivity strength between brain regions including FC, SC, gFCD, lFCD, EC, and ReHo.

Functional Connectivity (FC) among brain regions indicates its correlation with brain activity (Liu et al., 2023). The value of FC is transformed into a z-score through Fisher’s r-to-z transformation of the Person correlation coefficient between the function of a single brain area (reflected in the stable peak of EEG or MEG or the BOLD value of MRI) and all other brain area functions in the time domain (Wetherill et al., 2015). Given that the brain activity in patients with JME is based on normal brain activity, the FC in the healthy control group should be close to 0 such that a larger absolute value of FC corresponds to a greater difference in functional connectivity strength between patients with JME and the healthy control group.

The algorithm for calculating the structural connectivity (SC) values is the same as that for FC to calculate the functional connectivity strength between a single brain region and all other brain regions; however, the data comes from a single brain region and its structurally adjacent brain regions rather than all the other brain regions (Wang Y. et al., 2022). This means that as compared to FC, SC better reflects the strength of functional connections between the local brain regions (Liégeois et al., 2020).

Moreover, the global functional connectivity density (gFCD) calculates the ratio of FC values to the voxels in different brain regions, eliminating the impact of differences in the brain area volume on FC (Pan et al., 2022). The calculation method for local functional connectivity density (lFCD) is similar to that of gFCD, which represents the ratio of SC values to voxels in the brain regions (Jia et al., 2018).

Effective Connectivity (EC) refers to FC in the brain region corrected for multiple jackknife sensitivity analyses (Varangis et al., 2021). Compared to FC, EC has higher specificity but lesser sensitivity.

Regional Homogeneity (ReHo) is the Kendall coefficient between the function of different brain regions under time ranking, which reflects the regional synchronizations in different brain regions over the time domain (Jin et al., 2017). At present, high ReHo is considered to be one of the characteristics of resting state MRI in healthy individuals (Wang L. et al., 2022).

The algorithm for degree centrality (DC) is shown in Equation (1) as follows:


(1) $$C_{D}\left(N_{i}\right)=\sum_{j=1}^{g}x_{i\,j}\left(i\neq j\right)$$

Degree centrality refers to the total number of direct connections between node i and other g-1 nodes (Chang et al., 2022). In this study, DC refers to the number of brain regions that have direct functional connections to other brain regions, and it is the most intuitive indicator of the importance of brain regions.

The algorithm for mediating centrality is shown in Equation (2):


(2) $$C_{B}\left(N_{i}\right)=\sum_{j,k=1}^{g}sd\left(j,i,k\right),\left(j\neq k\right)$$

Here, sd (j, i, k) indicates that the shortest path from j to k that passes through i, i.e., i is on the shortest path from j to k (Samandari Bahraseman et al., 2022). CB reflects the importance of nodes in the network structure. The greater value of CB corresponds to greater significance of the node in maintaining the network topology. This study was conducted based on the postulation that abnormal firing of brain regions can affect the surrounding brain regions via white matter fibers, which further affects the functional network structure of the whole brain. Therefore, with a greater value of CB in the brain region, it is more likely that the abnormal discharge originating from this node will cause patients with JME to transition from a resting to an epileptic seizure.

Finally, we included the aforementioned quantitative indicators that could reflect the FC in brain regions and converted them into two categories of qualitative indicators, based on the increase or decrease of FC in brain regions in patients with JME as compared to healthy controls.

### 2.2. Network analysis

The Label Propagation Algorithm and spring layout were used in this study to assign each node in the small-world network model to different communities (Chin and Ratnavelu, 2016). Nodes in the same community implied that they were governed by the same core network. The colors of the nodes in the same community were consistent. In this study, we calculated the CB and CD of each node using equations (1) and (2). To display the information contained in the network more intuitively, we used NetworkX, Matplotlib, and NumPy in Python for this study to visualize the small world model, where the size of nodes was proportional to the centrality of the node mediations. We used two node reduction methods to highlight the core nodes of the network. When the node reduction method was used for the first time, the model no longer calculated the value of CB of the termination node, which refers to a node that could not affect the other nodes. This implied that the nodes with radii in the network could affect the network structure. The results are shown in the section “3.2.2. Brain functional connectivity network in patients with JME during resting state” During the second time, the node reduction method was used to filter out nodes with a degree greater than 10 and to reconstruct a small-world network model. Refer to Supplementary Material for specific codes and key parameters.

## 3. Results

### 3.1. Summary of data

**FIGURE 3:**
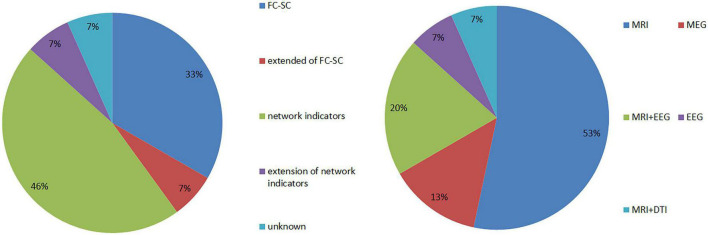
Summary of research equipment and research indicators.

A total of 15 articles were included in the study, which comprised 462 patients with JME and 387 patients in healthy control groups. Further, they involved five types of equipment combinations, including MRI, MEG, EEG, MRI+EEG, MRI+DTI, and involved five major categories of research indicators, including FC-SC; extended indicators of FC-SC (lFCD, gFCD, EC); network model indicators (global efficiency, local efficiency, effective connectivity); extension of network model indicators (Reho); and unknown statistical indicators (see Figure 3 for more details).

### 3.2. Model construction

#### 3.2.1. Whole brain functional connectivity network mode of patients with JME in the resting state

**FIGURE 4:**
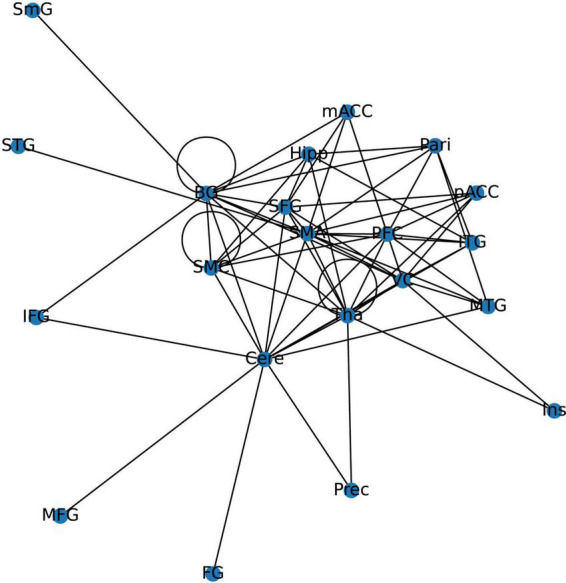
Brain Area Network Connection Network. SmG, supramarginal gyrus; STG, superior temporal gyrus; MTG, middle temporal gyrus; ITG, inferior temporal gyrus; FG, frontal gyrus; IFG, inferior frontal gyrus; SFG, superior frontal gyrus; PFC, prefrontal cortex; Prec, pericalcarine cortex; Ins, insula; Pari, orbitofrontal cortex; pACC, posterior cingulate cortex; mACC, middle cingulate cortex; BG, basal ganglia; Hipp, hippocampus; VC, visual cortex; Tha, thalamus; SMC, primary motor sensory cortex; SMA, supplementary motor area; Cere, cerebellum.

The 21 nodes in the graph represent 21 different brain regions included in the study, and the edges represent the presence of FC between two nodes in a given network. The distribution of various brain regions in the functional connectivity network is shown in Figure 4, 5. The CB ranking of each functional brain area and the number of brain areas with the same degree are shown in Figures 6, 7.

**FIGURE 5:**
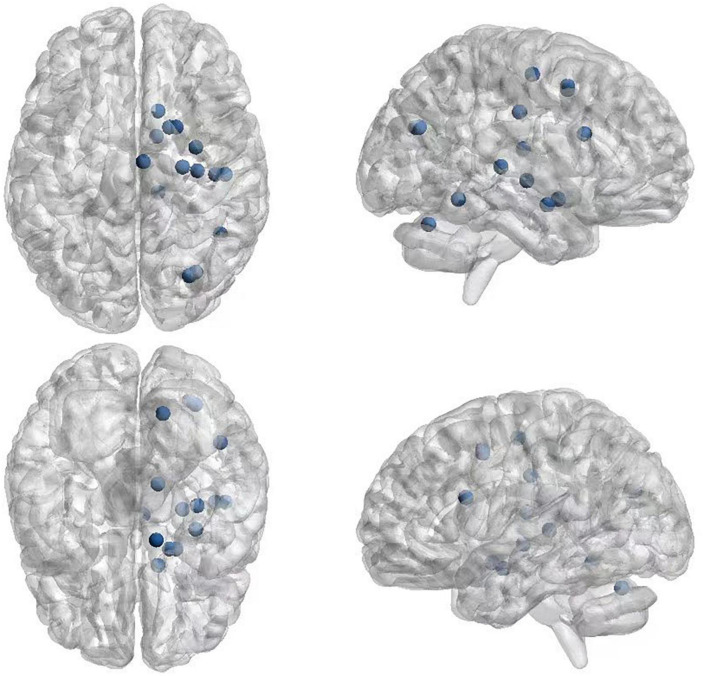
Brain Area visualized on top of a brain template.

**FIGURE 6:**
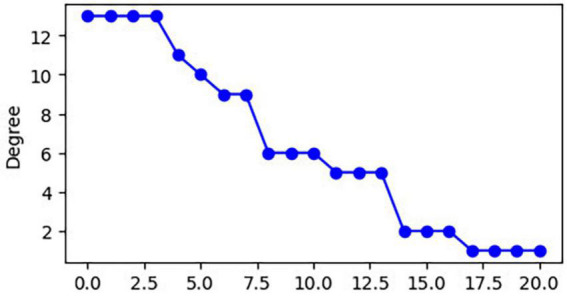
Rank map of the degree of functional brain regions.

**FIGURE 7:**
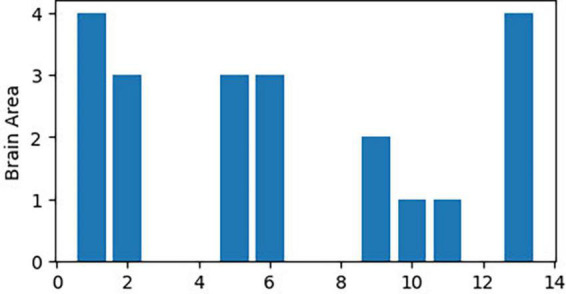
Functional brain area distribution histogram of degree.

#### 3.2.2. Brain functional connectivity network in patients with JME during resting state

From the original code, it can be observed that the indicators of LPA are transmitted to the color of nodes–the same node color indicates that they belong to the same community and are regulated by the same core network. In this study, the color of each node was displayed in purple, indicating that the 21 functional brain regions of patients with JME are regulated by the same core network in the resting state.

From the original code, it can be seen that the value of CB is proportional to the radius of the nodes in the graph, which implies that the size of the nodes in the graph is positively correlated with the CB of the brain region. The three blue dots with different radii shown in the legend represent node radius sizes of 1, 0.5, and 0.1 for CB.

Using the first node reduction method, we found that patients with JME have 21 different brain functional connectivity networks in the resting state. After running the code 500 times, we observed that these 21 different types of connection networks were distributed between (Abe et al., 2007; Vollmar et al., 2012; Chin and Ratnavelu, 2016; Poleon and Szaflarski, 2017; Lee and Park, 2019; Qin et al., 2019; Ma et al., 2022; Samandari Bahraseman et al., 2022; Huang et al., 2023; Ke et al., 2023). As the number of runs increased, the probability of each type of connection network appearing gradually approached 1/21.

According to the differences in the size of CB in different brain regions, 21 functional connectivity networks could be roughly divided into three categories.

**FIGURE 8:**
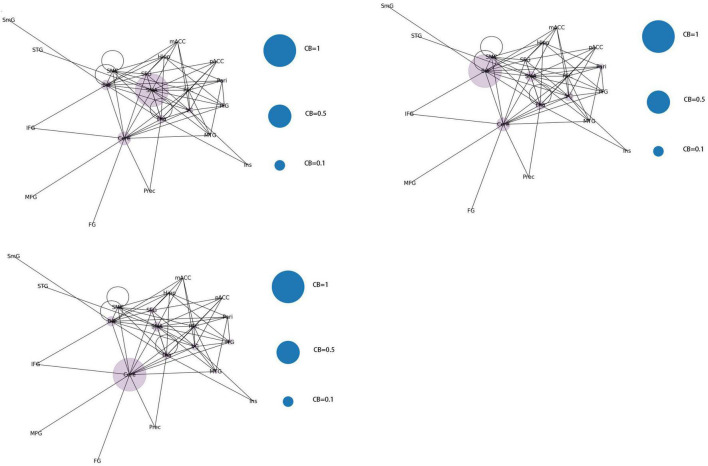
Whole brain functional connectivity patterns with increased CB in individual brain regions.

Category 1 (Figure 8): Significant increase in CB in a single brain area (CB > 0.8) with three scenarios: SMA, Cere, and BG.

**FIGURE 9:**
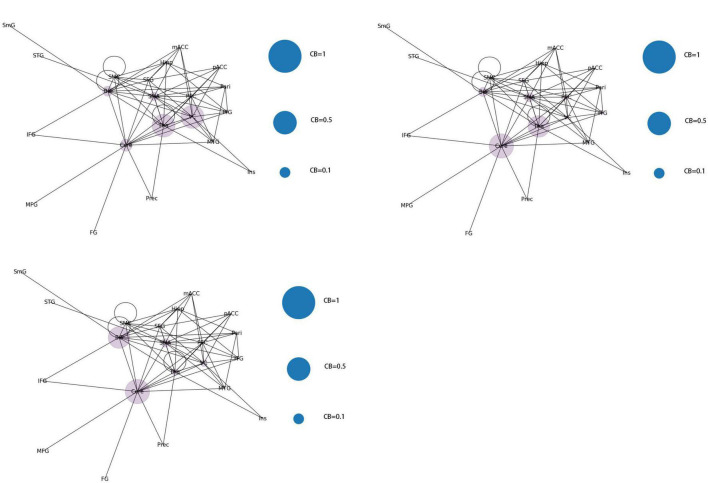
Whole-brain functional connectivity pattern with increased CB in two brain regions.

Category 2 (Figure 9): Two brain regions showed a significant increase in CB (0.5 < CB ≤ 0.8) with three scenarios: Tha-VC, Cere-Tha, and Cere-BG.

**FIGURE 10:**
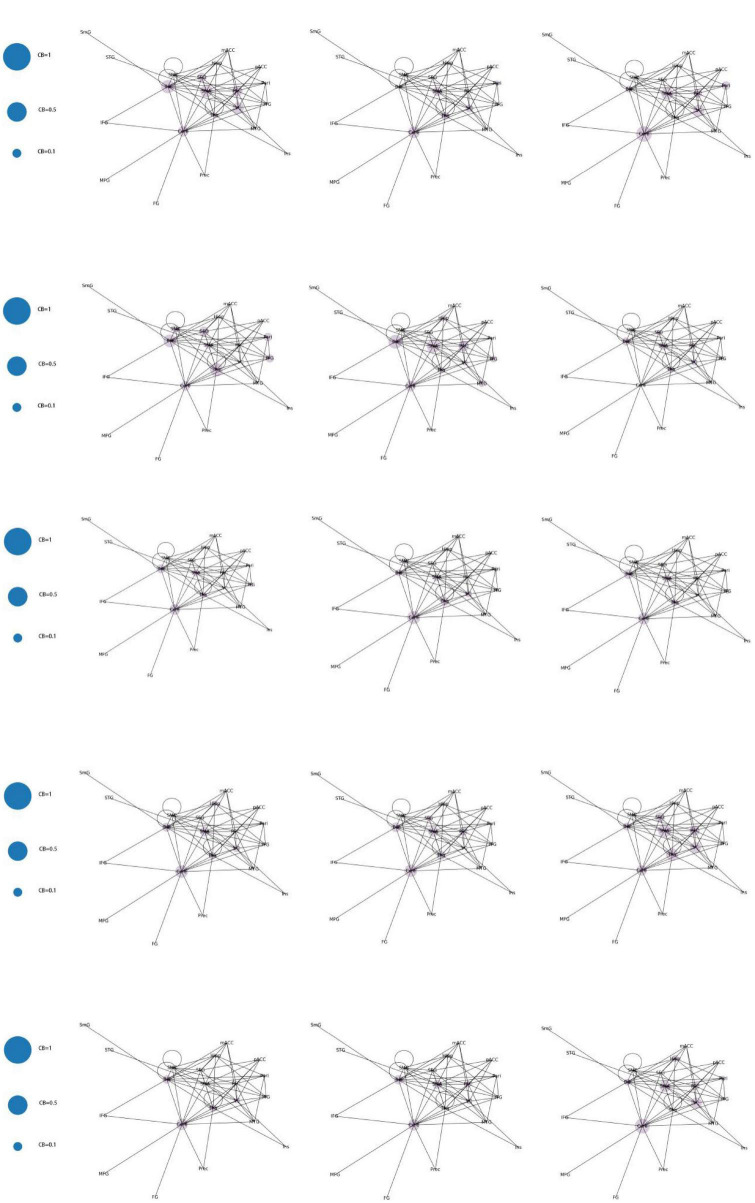
Whole brain functional connectivity without central increase mediated by brain regions.

Category 3 (Figure 10): No significant increase in mediating centrality was observed in the remaining 15 images (CB < 0.5).

### 3.3. Functional connectivity based on topological brain regions

**FIGURE 11:**
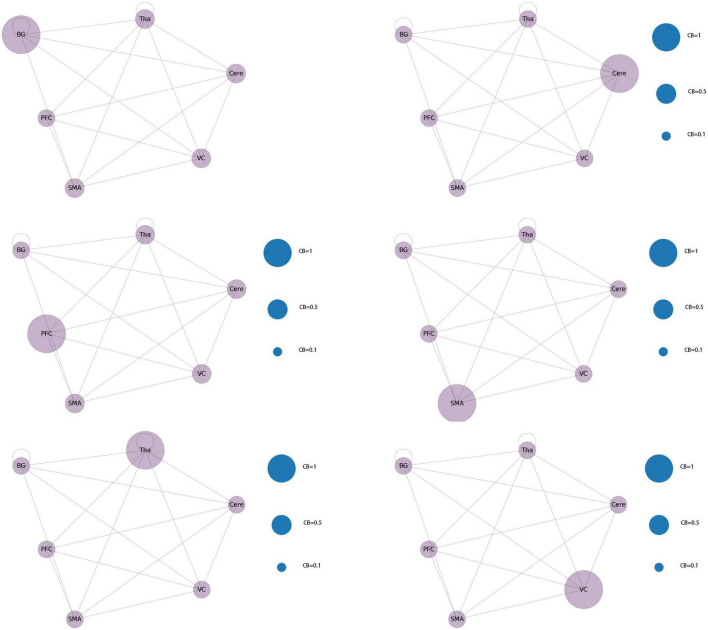
Core brain area functional connectivity network mode.

After the second node reduction, the results revealed that six brain regions, namely, Tha, Cere, BG, SMA, VC, and PFC were the core regions of abnormal functional connectivity in patients with JME during the resting state. Six different functional network connectivity patterns were discovered by constructing functional connectivity networks from six core brain regions. The result is shown in Figure 11. After running the code 100 times, the probability of each type of connection network appearing gradually approached 1/6.

### 3.4. Simplified functional connection network based on white matter fiber tracts

A previous study found that anatomical fiber tracts underlie functional synchronization in white matter (Huang et al., 2023). The study further incorporated the tracts of curving and projection fibers of the core brain region in the functional connectivity network composition. With the introduction of anatomical relationships, the abnormal activation of brain regions has a temporal sequence relationship. Further, the conclusions about changes in functional connectivity of brain regions with direct fiber connections included in this study should be independent. Therefore, the simplified functional connectivity network should meet the requirement that if there is a direct fiber connection between brain regions, the FC changes in the brain regions are also independent. For example, assuming that there is a direct fiber connection between the three brain regions A → B → C, if FCa-b is positive and FCb-c is negative, then FCa-c should be negative.

**FIGURE 12:**
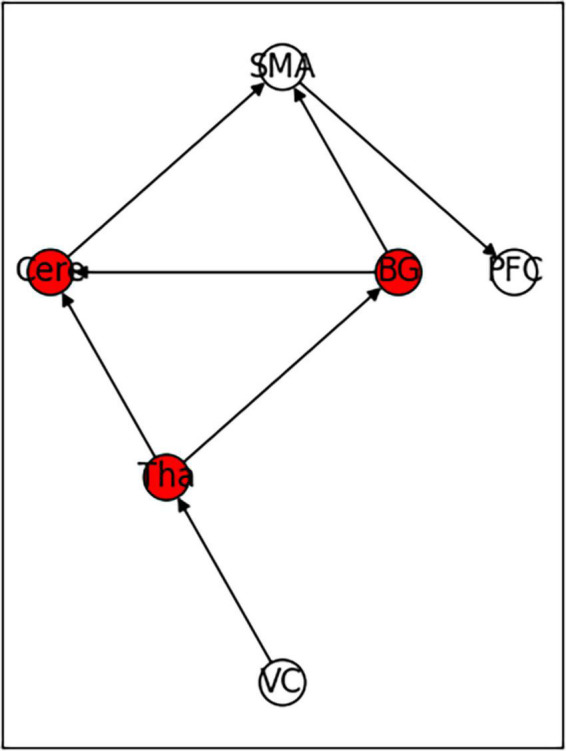
Functional connectivity model constructed based on the independence test of white matter fiber routing and functional connectivity.

According to the conclusions of previously screened papers, there were a total of 17 changes in functional connections that involved six core regions of the brain. After excluding two study conclusions that reported conflicting FC changes in the cerebellum and prefrontal cortex, six studies remained which reported increased FC, and nine studies with decreased FC in the brain regions. We used the aforementioned data to reconstruct a new small-world network. The functional connection network ultimately obtained was based on simplified white matter fiber tracks and met 13 changes. The only changes in functional connectivity between Cere and VC that were enhanced (with a positive FC value) and PFC and SMA connectivity that were weakened (with a negative FC value) were not met. The functional connection network is shown in Figure 12.

The average value of CB of the six core brain regions in the network were 0.33 for VC and PFC; 0.6 for CB of BG, Cere, and Tha; and 0.63 for SMA.

## 4. Discussion

By constructing and analyzing a small-world network model, this study was the first to report that patients with JME have 21 different brain functional connectivity networks in their resting states, which have no significant statistical difference in their distribution. For the first category of 21 different brain functional connectivity networks, SMA, BG, and Cere had a significantly increased CB in the functional connectivity network, implying that abnormal discharges originating from the three brain regions could significantly impact the structure of the global brain functional connectivity network, i.e., the patients with JME in the resting state were more likely to undergo seizures. For the second category, two brain regions (Tha-VC, Cere-Tha, and Cere-BG) showed a significant increase in CB. Compared to the first category, the second one showed more network stability. Further, in the third category, no brain area showed a significant increase in CB in the network. Based on the principle of CB discussed in sections “2.1. System mapping” and “3.2.1. Whole brain functional connectivity network mode of patients with JME in the resting state,” we could determine that the stability of the rest of the 15 networks was higher in the first category than in the first category and the second category. The aforementioned result highlights that the functional connectivity and node importance of the brain regions of patients with JME changed dynamically in the resting state. Considering that seizures did not occur in patients with JME during the resting state, the study conjectured that analyzing the relationship between 21 different functional connectivity networks in the resting state and epilepsy symptoms in patients with JME is not meaningful. However, network analysis shows that the brain functional connectivity network of patients with JME is always controlled by the same set of core networks and that there is no significant difference in the distribution of the 21 brain connectivity networks. Therefore, the study presumed that patients with JME may have the following process in a resting state: the brain activity of patients with JME oscillates back and forth in brain functional connectivity networks. Under specific conditions, such as abnormal discharge caused by light stimulation and increased cognitive load, patients with JME may exhibit epileptic symptoms when their functional connection network had a weakened ability to resist abnormal discharge.

Using the rank map of the degree of functional brain regions and the functional brain area distribution histogram of degree (Figures 6, 7), we demonstrated that the importance of each brain region in the different networks is not consistent, as some brain regions concentrate a large number of functional connections. Combining the node reduction methods, we found that abnormal discharges originating from six brain regions i.e., Tha, Cere, BG, SMC, VC, and PFC are most likely to lead to epileptic seizure symptoms in patients with JME during resting state. The conclusion in the current study is the same as that of the anterior study, but the former is more comprehensive (Vollmar et al., 2012; Lee and Park, 2019; Qin et al., 2019; Ma et al., 2022). After incorporating the independence of white matter fiber tracts and brain interval FC in the network analysis, we found that the core network also had a certain ability to resist abnormal discharges due to their dynamic change and that the three subcortical brain regions, namely, Tha, Cere, and BG formed a highly coupled subnetwork of functional connections. We also found that the CB of VC and SFG in the core brain functional network was lower than that of the other four brain regions, indicating that abnormal discharge originating from VC and SFG had a smaller impact on the entire brain functional connection network, and thus the likelihood of seizures was lower. This explains the question that the erstwhile study could not explain, as to why only some patients with JME experience symptoms of reflex epilepsy due to light stimulation or increased cognitive load. Meanwhile, this also explains why only approximately 30% of patients with epilepsy in the cross-sectional study JME population have photosensitivity (Poleon and Szaflarski, 2017), while more than 90% of patients with JME experience seizures when targeted light stimulation is used (Ke et al., 2023). Patients with JME in the resting state have 21 different functional networks; however, only a portion of networks have VC with a large value of CB. For patients with JME with repeated light stimulation, the possibility of abnormal discharge originating from the VC and involving the whole brain functional connectivity network increased significantly, and this was close to 100%.

In addition, this study reported that the core brain functional connectivity network can meet the independence of the vast majority (88.2%) of FC; however, it could not meet the FC enhancement between Cere and VC and the FC attenuating between PFC and SMA exhibited by patients with JME. Combined with the special status of SFG and VC for cognitive function (Abe et al., 2007) and photosensitivity (Fisher et al., 2022), this study suggested that the changes in functional connectivity of Cere and VC and that of SFG and SMA were characteristic of the brain region functional connectivity in patients with JME.

## 5. Limitations

Considering the lateralization of the human brain function, most of the main functions are concentrated in one hemisphere (Güntürkün et al., 2020). Hence, in the process of experimental design, there was no conflict between the left and right hemisphere brain regions in the performance of functional connectivity. This consideration was confirmed in subsequent data collection when the functional connectivity of the same brain regions in the left and right hemispheres was compared with only two results i.e., the same changes in both the brain regions or the same changes in one brain region and no changes in the other as compared to that in healthy controls. This implied that if meaningless connections were added to the model to compare the differences between the two brain regions, systematic errors would be larger in the calculation of CB.

In the process of building the model, four brain regions were combined based on similar anatomical positions and similar functions, i.e., the parietal lobule and inferior parietal lobule were combined into the parietal lobe and the anterior central gyrus, posterior central gyrus, posterior cingulate gyrus, and SMA anterior cluster were combined into the SMC. Further, the eight subregions of the thalamus were merged into the thalamus and the globus pallidus, putamen, and caudate nuclei were merged into BG. Consolidation between brain regions can reduce the difficulty of model establishment and operation. However, considering that although MNI or ALL localization studies were selected during the data acquisition process, the voxel size of the selected images varied among different studies, and excessive division of brain regions could increase the errors in this study. Meanwhile, the excessively detailed division of the brain regions could result in only some voxels producing functional connections in a relatively unified overall brain region (Sun et al., 2023). This would confuse the FC conclusions screening of the study. Therefore, the study ultimately chose to merge some brain regions that had similar morphology and function.

The *p*-value of the conclusion in this paper was 0.05^19, which is close to 0 and less than 0.01. Among them, the type I error tolerance for the papers included in this study was 0.05. A total of 15 papers were included in this study. Furthermore, this study used two methods to control the false positive rate. First, we selected the same baseline for inclusion in the study. Second, the studies included had statistical significance with *p* < 0.05, which implies that the *p*-value for FDR testing on the research conclusion would also be less than 0.05. However, the false positive conclusion of this study could not be eliminated, which means that a portion of healthy individuals with abnormal EEG activity were treated as intermittent adolescent myoclonic epilepsy patients. Hence, it can be stated that there are changes in the importance of the brain regions (mediating centrality) in the resting state fMRI of normal people.

## 6. Conclusion

As mentioned above, the three conclusions of this study can be summarized as follows: First, the functional connectivity and node importance of the brain regions of patients with JME changed dynamically in the resting state. Second, the abnormal discharges originating from Tha, Cere, BG, SMA, VC, and PFC were more likely to cause epilepsy symptoms in patients with JME in the resting state. Third, low CB of PFC and VC explained why light stimulation or increased cognitive load can induce epilepsy symptoms in only some patients with JME in the resting state.

## Data availability statement

The original contributions presented in this study are included in the article/Supplementary material, further inquiries can be directed to the corresponding author.

## Author contributions

DL: conceptualization, methodology, software, data curation, formal analysis, writing—original draft, and visualization. NZ: formal analysis, validation, data curation, and writing—review and editing. YL: validation, funding acquisition, and writing—review and editing. TW: conceptualization, methodology, software, data curation, formal analysis, writing—original draft, visualization, project administration, and funding acquisition. All authors contributed to the article and approved the submitted version.
