# Supplementary material for: Differences in the distribution of triggers among resting state networks in patients with juvenile myoclonic epilepsy explained by network analysis
Source: Front Neurosci. 2023 Oct 4;17:1214687. doi: 10.3389/fnins.2023.1214687 (PMC10582565; doi:10.3389/fnins.2023.1214687)
Supplement: Supplementary file 1 [file Data_Sheet_1.ZIP › Supplementary material/Date/新建 Microsoft Word 文档.docx]

2.2.1纳入分析的研究信息

1. 10.1111/ane.13079， [Lee](https://onlinelibrary.wiley.com/action/doSearch?ContribAuthorRaw=Lee%2C+Ho-Joon)等招募了36名drug-naïve的JME患者和30名健康对照组，通过MRI、EEG的手段，比较脑区及脑区间二者功能连接的差异，发现JME患者在整体连通性方面，与健康对照组相比，JME患者的整体效率、局部效率和小世界指数显著降低，而特征路径长度增加。而局部连通性方面左尾前扣带皮层、左中央前皮层、左顶叶上皮层和右前额上皮层的中介中心性增加，而海马的中介中心性降低。

2. 10.1016/j.eplepsyres.2020.106324，[Routley](https://pubmed.ncbi.nlm.nih.gov/?term=Routley+B&cauthor_id=32335503)等26名JME患者和26名健康对照组，通过MEG的手段，比较二者脑脑区及脑区间功能差异，发现JME右感觉运动皮层的连通性下降。而右颞下回和右前叶、右颞下回和右顶叶下回、右角回和右楔前叶以及左缘上回和左角回，以及右中央前回和右中央后回之间连通性减少（FC是指时域中MEG最大振幅之间的相关性）

3. 10.1155/2018/7392187，[Zhong](https://pubmed.ncbi.nlm.nih.gov/?term=Zhong%20C%5BAuthor%5D)收集了25名JME患者和24名健康对照组，通过rs-fMRI的手段，比较二者脑区间功能的差异，发现

·右前小脑与左侧小脑后回、右侧枕中回、左侧枕上回和双侧中央后回的FC增加，与左侧额上回、双侧眶额下回、左侧颞中回和左侧SMA的FC减少。

·左前小脑与左侧枕中回簇、右侧枕上回簇和双侧小脑前叶的FC增加，与右额中回、右SMA、左眶额下回和左额中叶的FC减少。

·小脑蚓与左侧颞中回、左侧梭状回和右侧楔前叶的功能连接增加，与左侧SMA的功能连接减少。

·左侧壳核与右海马和左中扣带回的FC增加，双侧SMA下回、右中央前回、双侧顶上小叶和左顶叶下叶的FC减少。

·左侧尾状核与右侧小脑下叶和左侧缘上回的FC增加，左侧眶上额叶和左侧中央旁小叶的FC减少。

·右侧尾状核与双侧小脑后部和双侧尾状核的功能连接增加。

·右眶额上回和左中央后回也发现与右尾状体的功能连接减少。

·右侧丘脑与右后小脑和双侧丘脑的功能连接增加，与右额上回和左额中上回的功能连接减少。

·右侧眶额上回的与双侧脑岛回和双侧扣带中回的FC增加，右侧小脑前回、右侧颞下回、左侧颞中回、小脑蚓、右侧额上回内侧的FC减少。

·左侧颞中回与双侧颞上回簇、左侧顶叶下小叶簇和右侧中央后回簇的FC增加，左颞中回、右前小脑、左楔前小叶和左内侧上额叶FC减少。

·右侧海马体与左侧颞下回和双侧额中上回的FC增加，右侧颞下回、左侧中央后回、右侧Rolandic操纵盖和双侧枕顶小叶的FC减少。

4. 10.3390/brainsci12121658. [Ma](https://pubmed.ncbi.nlm.nih.gov/?term=Ma%20L%5BAuthor%5D)等人招募了34名JME患者和34名健康对照组，通过fMRI手段，比较脑区及脑区间二者功能连接的差异，发现JME患者在左小脑后叶（CePL.L）、右颞下回（ITG.R）和右额上回（SFG.R）的DC显著增加，左额下回（IFG.L）和左颞上回（STG.L）的DC降低。JME患者还显示从小脑区域到大脑区域的EC单向增加，包括从CePL.L到右楔前叶（PreCU.R），从左小脑前叶（CeAL.L）到ITG.R，从右小脑后叶（CePL.R）到IFG.L，以及从小脑左下半月小叶（CeISL.L）到SFG.R。

5. 10.1142/S0129065717500344 [Jiang](https://pubmed.ncbi.nlm.nih.gov/?term=Jiang+S&cauthor_id=28830309) [等人](https://pubmed.ncbi.nlm.nih.gov/28830309/#affiliation-1)招募了22名JME患者和25名健康对照组，通过rs-fMRI的手段，比较脑区及脑区间的二者功能连接的差异，发现：

·与健康对照组相比，JME患者在所有丘脑亚区岛叶和枕中回的FC显著增加

·楔前叶与所有丘脑细分之间的连通性显著降低。

·在JME中，除了右外侧前细分(组8)外，壳核与7个丘脑细分之间的连接，以及除了腹侧中间和右侧前细分(组3和组8)外，苍白球与6个丘脑细分之间的连接更强

·超过一半的丘脑碎片(组1、组3、组4、组5、组6和组7)显示出与背侧前扣带皮层的连通性增加。

·额叶的5个丘脑子区域以及颞叶的4个子区域也发现FC下降，在丘脑的五个亚区域也发现了FC的降低。

·左侧角回与两个丘脑亚区域的连通性减少:左侧外侧丘脑背侧(组4)和中后丘脑背侧(组7)。右侧内侧丘脑背侧(组1)和后丘脑(组5)分别与海马体和旁海马体的FC减少。

6. 10.1016/j.clineuro.2020.106119. [Kim](https://pubmed.ncbi.nlm.nih.gov/?term=Kim+J&cauthor_id=32763668)通过招募38名药物抵抗的JME患者和40名健康对照组，通过EEG的手段，发现AED不良反应的JME患者相较于健康对照组全局效率、局部效率而小世界指数上升。JME患者包括药物抵抗的JME患者相较于健康对照组没有发现有效连接的改变。

8. 10.1212/WNL.0b013e3182563b44 [Vollmar](https://pubmed.ncbi.nlm.nih.gov/?term=Vollmar+C&cauthor_id=22551729)等人招募了29名JME患者和28名健康对照组，通过fMRI和DTI的手段，发现SMA前簇显示出与前额叶和额颞叶区域的连接减少，与中央区域和下行运动通路的连接增加。SMA簇显示与初级运动皮层的连接减少，与枕叶和侧颞新皮质的连接增加。前额叶认知皮层和运动皮层之间的结构连接增加，前额叶和额颞叶区域之间的连接减少，枕叶皮层和SMA之间的连接增加。

9. 10.3389/fneur.2018.00838.Jia等人招募了32名JME患者和60名健康对照组，通过fMRI等手段，发现与健康对照组相比，IGE与gFCD位于左上顶叶回，显着减少的gFCD位于顶叶的左下外侧其余部分，后部的左扣带回和右上额回。lFCD显着增加位于左上顶叶回，lFCD显着减少位于右上顶叶回和左下外侧顶叶。IGE的双侧中央前回和中央后回中观察到偶联减少，与HC中的偶联相比。JME患者在小脑和双侧枕中回中的偶联增加。与HCs组相比，JME组显着增加的gFCD和lFCD位于左上顶叶回中。

10. 10.1016/j.nicl.2014.11.018. Caeyenberghs等招募了34名JME患者和匹配的健康对照组，通过MRI的手段，发现与健康对照组相比JME患者显示出小世界值与健康对照没有差异，与对照组相比，JME患者左侧中央后回的节点效率显着增加。该子网包括主要运动区域（左中央前回），顶叶皮质区域（双侧中央后回，右前突），皮质下区域（左壳，左苍白球），左小脑小叶IV-V和右海马。涉及的皮质和皮质下区域如图2所示。与对照相比，所有连接在患者中表现出增加的值。

11. 10.1111/epi.12580. [Jung Bin Kim](https://onlinelibrary.wiley.com/action/doSearch?ContribAuthorRaw=Kim%2C+Jung+Bin)等招募了31名JME和42名健康对照组，通过MRI的手段，FC发现与健康对照组相比，JME患者内侧前额叶皮层MPFC和楔前/后扣带皮层（PCC）的FC显著降低；JME患者（n=31）的右侧MPFC显著降低。

12. 10.1016/j.nicl.2019.101759. Qin等招募了18名JME患者，通过EEG和MRI等方法，发现与高EEG网络变化高度相关的阳性BOLD反应主要位于感觉和运动区域，即中扣带皮层（MCC）、补充运动区（SMA）、中央旁小叶以及小脑、楔前叶和颞下叶，丘脑中的BOLD反应呈阳性，少量的尾状体和双侧脑岛。相反，在双侧额叶、前扣带皮层（ACC）、中央后区、颞中区、顶叶下区和枕叶区发现了与中等EEG网络变化相关的阴性BOLD反应。

额叶区域和ACC与SMA、中央旁小叶区域的功能耦合减弱，小脑与额叶（额叶上和额叶内侧）和感觉运动相关（SMA和中央旁小叶）区域的功能偶联减弱。此外，丘脑与额叶区域的连通性降低。额叶区域（frontal_sup、frontal_medial和ACC）内的功能耦合增加，以及从额叶区域到小脑的连接增加。此外，调节相互作用分析表明，丘脑、小脑、额叶和感觉运动相关区域主要存在定向负调节效应。

13. 10.1016/j.seizure.2020.09.022. [Dong Ah Lee](https://pubmed.ncbi.nlm.nih.gov/?term=Lee+DA&cauthor_id=33045541) 等招募了56例JME患者，通过EEG和MRI等方法，发现在JME患者中，全局效率、局部效率和平均聚类系数显著降低，而特征路径长度和小世界指数增加。在JME患者中，右半球的额前中皮质和额后中皮质，以及额前中、后扣带回、枕外侧和海马旁皮质以及左半球的楔前叶被确定为中枢节点。在JME患者中，与健康对照组相比，F4、F7、F8、Fp1和Fp2电极的介数中心性增加。

14. 10.27204/d.cnki.glzhu.2022.001635 Han等招募了25名新诊断的JME患者和28名健康对照组，发现在全局属性中,JME患者大脑结构网络的全局效率及局部效率较HC组降低,特征路径长度增加;(2)在局部区域,相比HC组,JME患者的6个脑区的节点效率显著降低,包括右侧中央前回、右侧背外侧额上回、右侧后扣带回、右侧尾状核及双侧额中回,右侧后扣带回的节点效率及度中心性均显著降低。

15. 10.27005/d.cnki.gdzku.2021.000128 Jiang等招募了19名JME和22名健康受试，通过rsf-MRI，发现,JME患者双侧丘脑和运动相关皮质区域的局部一致性与健康对照相比存在显著升高,小脑和枕叶的局部一致性明显减小。

16. [10.3390/brainsci12121658](https://doi.org/10.3390%2Fbrainsci12121658) [Laiyang Ma](https://sciprofiles.com/profile/author/aEExRW5zaUJlLzBQbE9ibFVWVEUzNk5nVmpROWVsQzJCWUluTFpsMDNqcz0=)招募了34名JME患者和健康对照组，通过MRI等手段发现，与HC相比，JME患者左小脑后叶（CePL.L）、右颞下回（ITG.R）和右额上回（SFG.R）的DC显著增加，左额下回（IFG.L）和左颞上回（STG.L）的DC降低。患者还显示从小脑区域到大脑区域的EC单向增加，包括从CePL.L到右楔前叶（PreCU.R），从左小脑前叶（CeAL.L）到ITG.R，从右小脑后叶（CePL.R）到IFG.L，以及从小脑左下半月小叶（CeISL.L）到SFG.R。

感觉运动皮层

右前小脑与左侧小脑后回、右侧枕中回、左侧枕上回和双侧中央后回的FC增加，与左侧额上回、双侧眶额下回、左侧颞中回和左侧SMA的FC减少。

1左尾前扣带皮层、左中央前皮层、左顶叶上皮层和右前额上皮层的中介中心性增加，而海马的中介中心性降低。

2JME右感觉运动皮层的连通性下降

3.右侧颞中回活动增加

4. 在左小脑后叶（CePL.L）、右颞下回（ITG.R）和右额上回（SFG.R）的DC显著增加，左额下回（IFG.L）和左颞上回（STG.L）的DC降低

5. JME组显着增加的gFCD和lFCD位于左上顶叶回中。

6. JME患者左侧中央后回的节点效率显着增加

7. JME患者（n=31）的右侧MPFC显著降低。

8. 中扣带皮层（MCC）、补充运动区（SMA）、中央旁小叶以及小脑、楔前叶和颞下叶，丘脑中的BOLD反应呈阳性，少量的尾状体和双侧脑岛。相反，在双侧额叶、前扣带皮层（ACC）、中央后区、颞中区、顶叶下区和枕叶区发现了与中等EEG网络变化相关的阴性BOLD反应。

右半球的额前中皮质和额后中皮质，以及额前中、后扣带回、枕外侧和海马旁皮质以及左半球的楔前叶被确定为中枢节点。

9.JME患者的6个脑区的节点效率显著降低,包括右侧中央前回、右侧背外侧额上回、右侧后扣带回、右侧尾状核及双侧额中回,右侧后扣带回的节点效率及度中心性均显著降低。

发现,JME患者双侧丘脑和运动相关皮质区域的局部一致性与健康对照相比存在显著升高,小脑和枕叶的局部一致性明显减小。

10.JME患者左小脑后叶（CePL.L）、右颞下回（ITG.R）和右额上回（SFG.R）的DC显著增加，左额下回（IFG.L）和左颞上回（STG.L）的DC降低。

·小脑-枕叶增加

·小脑-额上回减少

·小脑-SMA减少

·基底节-SMA减少

·基底节-小脑增加

·丘脑-额上回减少

·小脑-额上回增加

·基底节-丘脑增加

·丘脑-枕叶减少

·视觉皮层-丘脑减少

·SMA-枕叶增加

·SMA-额叶减少

·小脑-视觉皮层增加

·基底节-小脑增加

·额上回-SMA减少

·小脑-额叶减少

·丘脑-额叶减少
