# Supplementary material for: Differences in the distribution of triggers among resting state networks in patients with juvenile myoclonic epilepsy explained by network analysis
Source: Front Neurosci. 2023 Oct 4;17:1214687. doi: 10.3389/fnins.2023.1214687 (PMC10582565; doi:10.3389/fnins.2023.1214687)
Supplement: Supplementary file 1 [file Data_Sheet_1.ZIP › Supplementary material/Date/检索式.docx]

MESH

JME：Janz Syndrome；Juvenile Myoclonic Epilepsy；JME；impulsive Petit Mal Epilepsy

functional connectivity

JME AND functional connectivity 72篇

(Juvenile Myoclonic Epilepsy[MeSH Terms]) AND (functional connectivity) 36篇

(Juvenile Myoclonic Epilepsy[MeSH Terms]) AND (functional connectivity[Title/Abstract])22篇

(Juvenile Myoclonic Epilepsy) AND (functional connectivity) 50篇

本文以“功能连接 and 青少年肌阵挛癫痫”和“灰质厚度 and 青少年肌阵挛癫痫”为关键词在CNKI，PubMed和Elsiver数据库刷选了97篇论文，最终纳入了19项研究，共JME患者397例，健康对照组408例。

然后文章通过MATLAB构建CRF模型，边的特征函数的权值由FC的数值限制，节点的特征函数由FC的CI决定，利用维特比算法分析青少年肌阵挛癫痫（JME）患者静息态功能连接（FC）与灰质厚度的关系。

结果：本文最后筛选了丘脑、小脑、额上回，基底节，辅助运动区和距状旁回6个灰质体积和功能连接同时改变的脑区，以这些脑区为最大团C，构建了CRF模型。

JME患者的基底节对丘脑起源的癫痫电活动存在解码作用

3.CRF解释了丘脑-基底节环路和丘脑-额上回环路上存在白质纤维互补，而JME肌阵挛比例远高于失神性发作的比例

4.模型预测了前庭神经核存在小脑有负的FC联系青少年肌阵挛癫痫（JME）患者静息态功能连接（FC）与灰质厚度的关系

5.
