# Supplementary figures and images for: Differences in the distribution of triggers among resting state networks in patients with juvenile myoclonic epilepsy explained by network analysis
Source: Front Neurosci. 2023 Oct 4;17:1214687. doi: 10.3389/fnins.2023.1214687 (PMC10582565; doi:10.3389/fnins.2023.1214687)

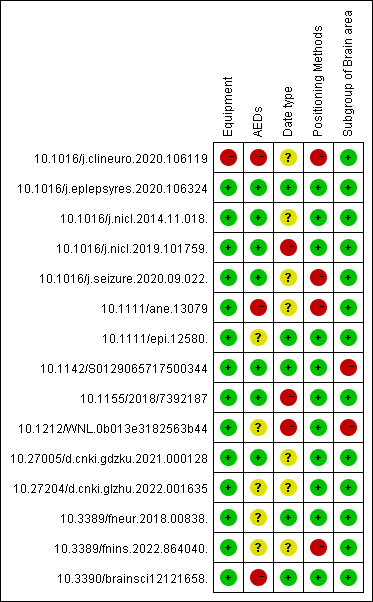

Supplement: Supplementary file 1 [file Data_Sheet_1.ZIP › Supplementary material/Code/Risk of bias summary.png]
